# Estimation of postmortem interval by vitreous potassium evaluation with a novel fluorescence aptasensor

**DOI:** 10.1038/s41598-017-02027-1

**Published:** 2017-05-12

**Authors:** Yanjun Ding, Xingmei Li, Yadong Guo, Weicheng Duan, Jiang Ling, Lagabaiyla Zha, Jie Yan, Ying Zou, Jifeng Cai

**Affiliations:** 1 Department of Forensic Science, School of Basic Medical Sciences, Central South University, Changsha, 410013 Hunan P.R. China; 2 Department of Pathophysiology, School of Basic Medical Sciences, Central South University, Changsha, 410013 Hunan P.R. China

## Abstract

Estimation of postmortem interval (PMI) is a central role in medico-legal identification. Analysis of vitreous potassium ions (K^+^) concentration is frequently used by forensic workers to estimate PMI. This paper describes interdisciplinary research to introduce fluorescence sensing techniques into forensic medicine. On the basis of silver nanoclusters (AgNCs) probe stabilized by DNA, a simple and highly sensitive fluorescence aptasensor has been proposed to selectively detect K^+^ ions. The linear range for K^+^ ions was found to be 0.1 nM-1 mM, with limit of detection of 0.06 nM. Moreover, 63 vitreous humour cases within 36 h after death were further studied to verify the utility of K^+^ ions in estimating the PMI. By the fluorescence aptasensor method, a new formula was built to determine the postmortem interval based on K^+^ ions concentration: PMI(h) = −0.55 + 1.66 × C_K_^+^(r = 0.791). And the real significance of this research was demonstrated by additional 6 cases with known PMIs. In comparison with the conventional method, the presented aptasensor strategy is cost-effective and easy in measuring vitreous K^+^, which may be potentially a better way for estimation of PMI in medico-legal practice.

## Introduction

The precise estimation of postmortem interval (PMI) is a difficult and challenging problem in forensic medicine and especially in medico-legal investigations. Previous studies have been carried out to estimate the PMI through death various metabolic, physiochemical and biochemical changes from various body fluids like blood, cerebrospinal fluid, and vitreous humour(VH)^1^. Postmortem vitreous humour, first described by Sturner in 1963^2^,because of its particular topography in a physically protected environment and resistance to microorganic contamination with bacterial degradation, provides a more suitable medium for biochemical postmortem analysis than cerebrospinal fluid and blood^3^. The biochemical components of VH like sodium, chloride, calcium, magnesium, phosphate and potassium have been reported to estimate PMI^4^. Investigations about vitreous sodium potassium ratio were reported in recent studies, but did not find any correlation between vitreous sodium-potassium ratio changes and postmortem interval^5^. Baniak *et al*.^6^ reported that sodium, calcium, and chloride levels have no role in estimating PMI, and Fang^7^ reported that PMI was not significantly correlated with vitreous Na^+^ ions and Cl^−^ ions concentrations. Of all the vitreous constituents, measurement of vitreous potassium is the most extensively studied parameter as a predictor of PMI^8, 9^.

Up to now, various analytical technologies have been utilized to detect vitreous K^+^ ions. For example, Bortolotti *et al*.^10^ reported capillary ion analysis combining UV detection which needed complex operating procedure and lack of sensitivity. Zhou *et al*.^11^ explored vitreous potassium measured by low pressure ion chromatography, yet the qualitative ability of the analyst was easily influenced by other components in the vitreous humour. Besides, a sequential injection system reported by Passos *et al*.^12^ could successfully detect both concentrations of K^+^ ions and hypoxanthine automatically, but it required complex pretreatments and long-running operations. Also, the ion-selective electrode method reported by Chandrakanth *et al*.^5^ needed regular replacement of the electrodes for measurement of vitreous K^+^ ions. The above-mentioned methods possess some advantages in the practical applications, but they are time-consuming processes and low efficiency. Accordingly, developing a new sensor method for detecting K^+^ ions in VH with rapidity and sensitivity is of considerable interest.

Currently, fluorescence sensing technology as a new and innovative strategy has been gained a great deal of popularity in various scientific fields, such as food safety^13^, medicine^14^, biological drug^15^, environmental monitoring^16^ and so forth. As a new class of fluorescent materials to construct a novel fluorescent sensing technique, silver nanoclusters (AgNCs) have been given prominent attention largely owing to their unique properties including nontoxicity, water-solubility and biocompatibility^17–19^. Over the years, various templates were used to synthetize AgNCs have been reported^20, 21^. Among these methods, single stranded DNA (ssDNA) as green-chemical template is now the focus of considerable interest for the synthesis and characterization of AgNCs^22, 23^. Aptamers are single-stranded nucleic acid ligands that offer a unique epitope with high affinity and specificity binding to specific target molecules^24^. Such aptamer-based sensor (aptasensor) possesses the advantages of low toxicity, green production and biocompatibility^25, 26^. The unique aptamer/AgNCs assembly was widely applied to serve as fluorescence probe and specific binding ligand, respectively. For example, Javanietal *et al*.^27^ explored the antimicrobial properties in bacteria employing the AgNCs stabilized by DNA. Notably, Ye *et al*.^28^ used the DNA-AgNCs to detect HIV-DNA sequences. Besides, a fluorescent aptasensor based on DNA-AgNCs for ochratoxin A detection was studied by Chen *et al*.^29^. To the best of our knowledge, the application of fluorescence sensing methods for detection of vitreous potassium and evaluation of the PMI is rarely reported in the forensic related literature.

Inspired by these pioneering research studies, in this work, we made attempts to develop a selective fluorescent sensing method for vitreous potassium detection by using DNA-encapsulated AgNCs as fluorescent probes and its further application to estimate PMI. Herein we design a novel self-assembly DNA sequence to synthesize AgNCs which can detect K^+^ ions in VH specifically. Consequently, according to the VH sample results obtained by the proposed fluorescence assay, a new formula about the relation between PMI and [K^+^] has been calculated. The developed fluorescence aptasensor can present the unique advantages of detection efficiency, high sensitivity, selectivity, and simple preprocessing.

## Materials and Methods

### Chemicals and apparatus

AgNO_3_ (99.99%), NaBH_4_ (98%) and KNO_3_ were purchased from Sigma-Aldrich. A novel DNA sequence of 5′-GGTTGGTGTGGTTGGATCCCCCCCCCCCC-3′ was synthesized by Shanghai Sangon Biotechnology Co. Ltd. (Shanghai, China). 5′-CCCCCCCCCCCC-3′(dC_12_) was the scaffold for synthesis of AgNCs and 5′-GGTTGGTGTGGTTGG-3′ was the K^+^ ions aptamer which was widely used in a great deal of researches^30, 31^. A concentration of 20 mM phosphate-buffered (PB) was used throughout the experiments. Other reagents were of analytical grade and used without further purification. All stock solutions were prepared with double-distilled water filtered by Milli-Q (Millipore, Billerica, MA).

All fluorescence measurements were recorded on a fluorescence spectrophotometer (Hitachi, F-4600) using a 350 μL quartz cell. The emission spectra of DNA-AgNCs were recorded from 400 nm to 750 nm. Both the emission and the excitation slits were set to 10 nM, the scanning speed was 240 nm min^−1^. The UV−vis absorption spectra were obtained on an UV−vis spectrophotometer (Shimadzu, UV-2450). The prepared DNA-AgNCs was analyzed by using a Titan G2 60–300 transmission electron microscope (TEM, FEI, USA). Estimation of the concentration of K^+^ levels was carried out using the automatic biochemistry analyzer instrument (Japan, HITACHI 7060).

### Preparation of vitreous samples

A prior approval was obtained from the Institutional Review Board (IRB) of Third Xiangya Hospital, Central South University to conduct this research. The autopsy cases were admitted to the Department of Forensic Science, Central South University. The methods were in accordance with the relevant guidelines and regulations regarding working with autopsy specimens as established by The IRB (number 2016-S043) of Third Xiangya Hospital, Central South University. Informed consent was obtained from next-of-kin relatives of the cases. PMIs were established from medico legal examiner and certified by local police departments. All the cases with known PMI within 36 hours were collected. Then the selected samples were excluded the obvious causes of death including septic shock, vitreous haemorrhage, metabolic syndrome and trauma to eye. Finally a total of 63 forensic cases (41 males and 22 females) were selected in this study and the age of the deceased ranged from 18.5 to 58 years with a mean of 42.5 years. Several scholars like Garget *et al*.^32^ and Swain *et al*.^4^ found no statistical male-female difference in the vitreous potassium, so it was unnecessary to divide the cases into different groups for statistics to be analyzed. Vitreous humour samples were collected, 0.2 mL clear vitreous humour was aspirated without exerting much pressure from outer canthus of each eye by using a sterilized 20-gauge hypodermic needle 1.5–2.0 ml crystal, and then centrifuged at 12000 rotations for 10 min. For detection in the fluorescence aptasensor system we designed, the supernatant of the vitreous humour samples were diluted to 1000 folds using PB buffer solution (0.02 mmol/L, pH = 7.0) and could be preserved in −20 °C and −70 °C in deep freezer respectively for delayed analysis within 24 h or long time later.

### Synthesis of DNA-AgNCs probes

DNA-stabilized AgNCs were synthesized according to the previous report with slight modification^33^. Briefly, 54 μL of 0.5 mM AgNO_3_ was mixed with 90 μL of 50 μM DNA (molar ratio of Ag^+^/DNA = 6:1) and stirred on a circular oscillator for 30 min. Next, 54 μL of 500 μm NaBH_4_ (molar ration of Ag^+^/NaBH_4_ = 1:1) was quickly added into the mixture to reduce Ag^+^ ions. The final solution was incubated at 4 °C in the dark for 3 h before use.

### Fluorescence detection of K^+^ ions using DNA-AgNCs probes

In this work, the fluorescence measurements of K^+^ ions were conducted using the synthesized DNA-stabilized AgNCs. After the preparation of DNA-AgNCs probes, a series of 50 µL of K^+^ ions solutions with different concentrations from 0.1 nM to 1 mM were added into 50 μL of DNA-AgNCs solution. The change of fluorescence intensity was calculated using the equation E = F − F_0_, where F and F_0_ refer to the fluorescence intensities of DNA-AgNCs (λ_em_ = 565 nm) in the absence and presence of K^+^ ions respectively. After reaction for 10 min at room temperature, the fluorescence intensity was detected under the optimal excitation wavelength of 565 nm. All samples were prepared in triplicate solutions and average values were obtained. Furthermore, the control tests for 10 μM of nitrates including Fe(NO_3_)_2_, Mg(NO_3_)_2_, Zn(NO_3_)_2_, Ca(NO_3_)_2_, Al(NO_3_)_3_, LiNO_3_, NaNO_3_ and NH_4_NO_3_ were conducted under the same conditions mentioned above. Subsequently, the results of probing K^+^ ions in real vitreous samples obtained by the developed method were compared with the traditional automatic biochemistry analyzer method. Furthermore, all statistical data of real vitreous samples were analyzed by SPSS, ver. 22.0, where the linear regression was applied to predict the PMI.

### Data Availability

The datasets generated during and/or analysed during the current study are not publicly available due to there is no corresponding database, but are available from the corresponding author on reasonable request.

## Results and Discussion

### Principle of K^+^ ions detection based on DNA-AgNCs probes

**Figure 1:**
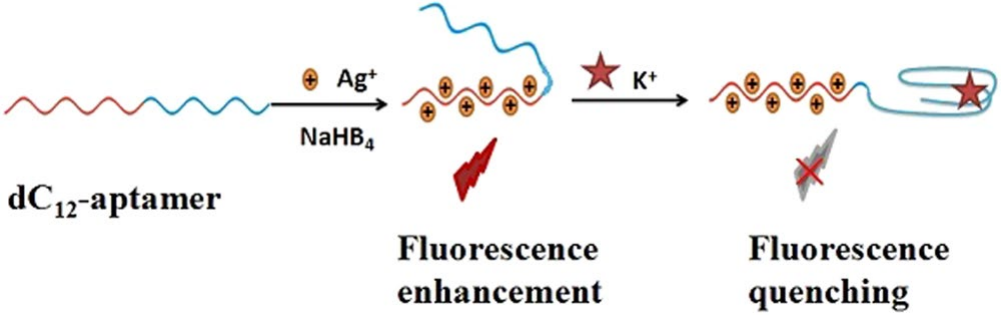
Schematic illustration of the DNA-AgNCs based fluorescence aptasensor assay of K^+^ ions.

The proposed fluorescence sensing mechanism for detection of K^+^ ions is shown in Fig. 1. In this work, a novel DNA template with two different nucleotide acid sequences was designed for the synthesis of the fluorescent DNA-AgNC sprobes. One specific sequence included GGTTGGTGTGGTTGG at the 5′-end, regarded as potassium aptamer fragment to identify potassium specially and form a guanine quartet^29, 34^, the other sequence was enriched with polycytosine oligonucleotide (dC_12_) at the 3′-end, used as a synthesis template for AgNCs to invigorate characteristic fluorescence signal. Besides, the 5′-end of proposed nucleic acid sequence enriched in ‘G’ may have affinity to the 3′-end enriched in ‘C’ through a Watson-Crick base-pairing interaction with a secondary structural transformation. Therefore, the synthetic novel DNA-AgNCs probes display strong fluorescence emission at 630 nm when excited at 565 nm. Whereas, once a solution containing K^+^ ions was introduced, the potassium could bind its aptamer specifically to form G-quadruplex structures and the secondary structure between 3′-end and 5′-end was transformed. These structural changes may induce greatly quenching the fluorescence of DNA-AgNCs. The concentration of the K^+^ ions can be detected quantitatively via the change of the fluorescence intensity detected by DNA-AgNCs.

### Characteristics of DNA-AgNCs probes

**Figure 2:**
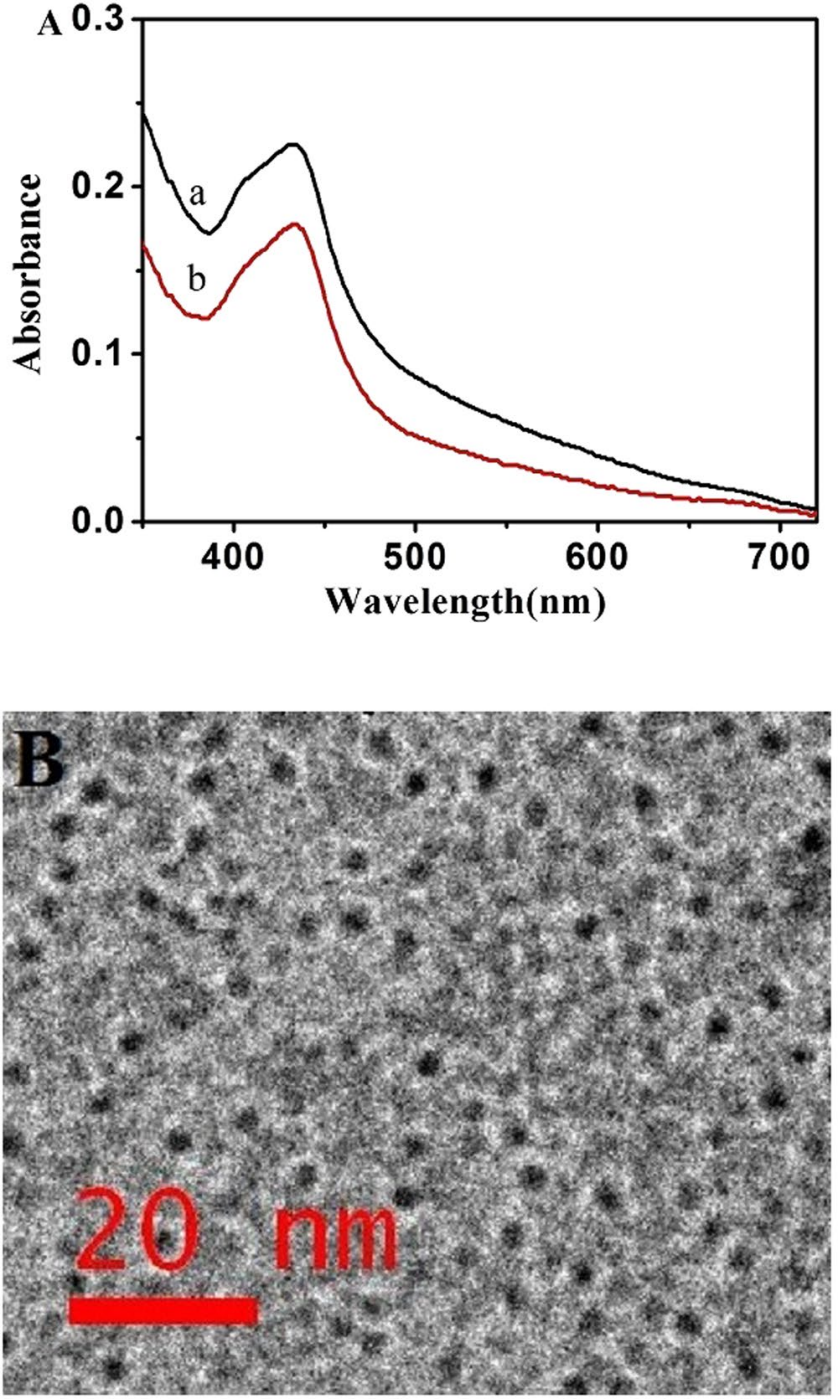
(**A**) UV−vis spectra of DNA-AgNCs in the absence and presence of 10 μM K^+^ ions. (**B**) TEM images of DNA-AgNCs.

To explore the mechanism of DNA-AgNCs as the model fluorescent probe, UV-vis spectra of DNA-AgNCs probes were recorded in the absence and presence of K^+^ ions. As shown in Fig. 2A (curve a), the DNA-AgNCs display an absorbance peak at 442 nm^23^. Interestingly, it is decreased after the exposure to 10 μM K^+^ ions (Fig. 2A curve b), indicating that there was an obvious response between the designed DNA-AgNCs and K^+^ ions. The change was mainly due to the aptamer part bounded to K^+^ ions and further folded to a G-quaduplex structure, via intramoleuclar hydrogen-bonding interactions^35^. The formation for DNA-AgNCs was further verified and characterized by TEM. TEM result showed that the as-prepared DNA-templated Ag NCs have an average size of 2–3 nm (Fig. 2B).

### Optimization of fluorescence response

**Figure 3:**
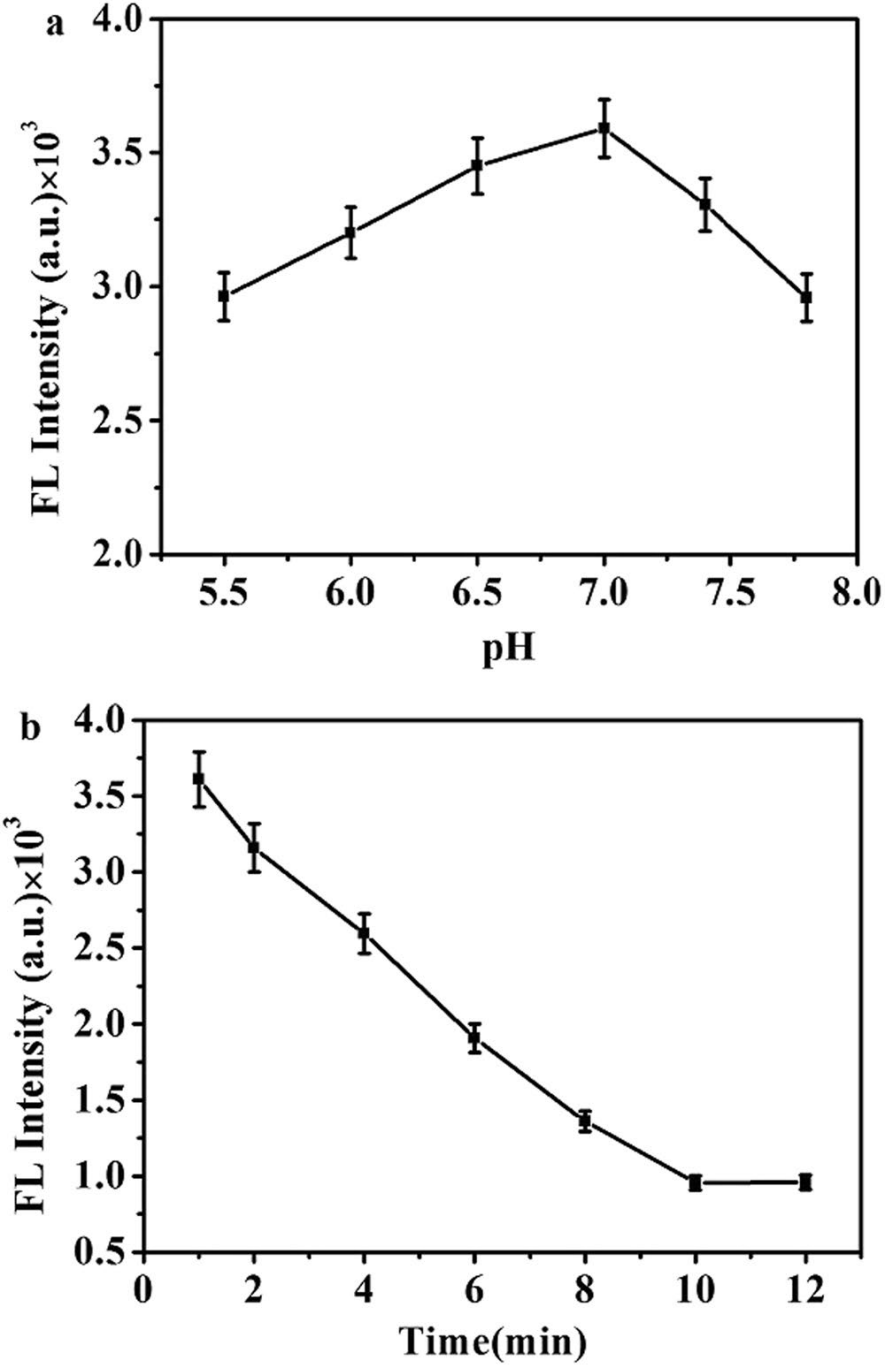
(**a**) pH dependence of fluorescence spectra of DNA-AgNCs under the following conditions: 10 μM K^+^ ions with varying pH values of PB(20 mM), incubated for 10 min at room temperature. (**b**) Time-dependent fluorescence intensity changes of the DNA-AgNCs for 10 μM K^+^ ions.

To study the optical performance of fluorescent DNA-AgNCs assays for the detection of K^+^ ions, the influences of pH value and incubation time in response system have been investigated. K^+^ activity and fluorescence intensity of DNA-AgNCs are relative to pH, as presented in Fig. 3a. It could be discovered that the fluorescence intensities of DNA-AgNCs increased gradually with the increasing value of pH from 5.5 to 7.0, beyond which fluorescence decreased, indicating that the pH 7.0 was the optimal one in this study. Figure 3b shows the experimental condition of reaction time-interval, fluorescence intensity of DNA-AgNCs was decreased gradually after the addition of K^+^. 50% of fluorescence reductions were observed during the 5 minutes. After 10 minutes, the reduction in fluorescence intensity dropped to the minimum, which indicates the fluorescence spectra reached the maximum quenching after a fixed time interval of 10 min. To obtain the best sensitivity, 10 min was selected as the optimal reaction time.

### Quantitative analysis of potassium ions

**Figure 4:**
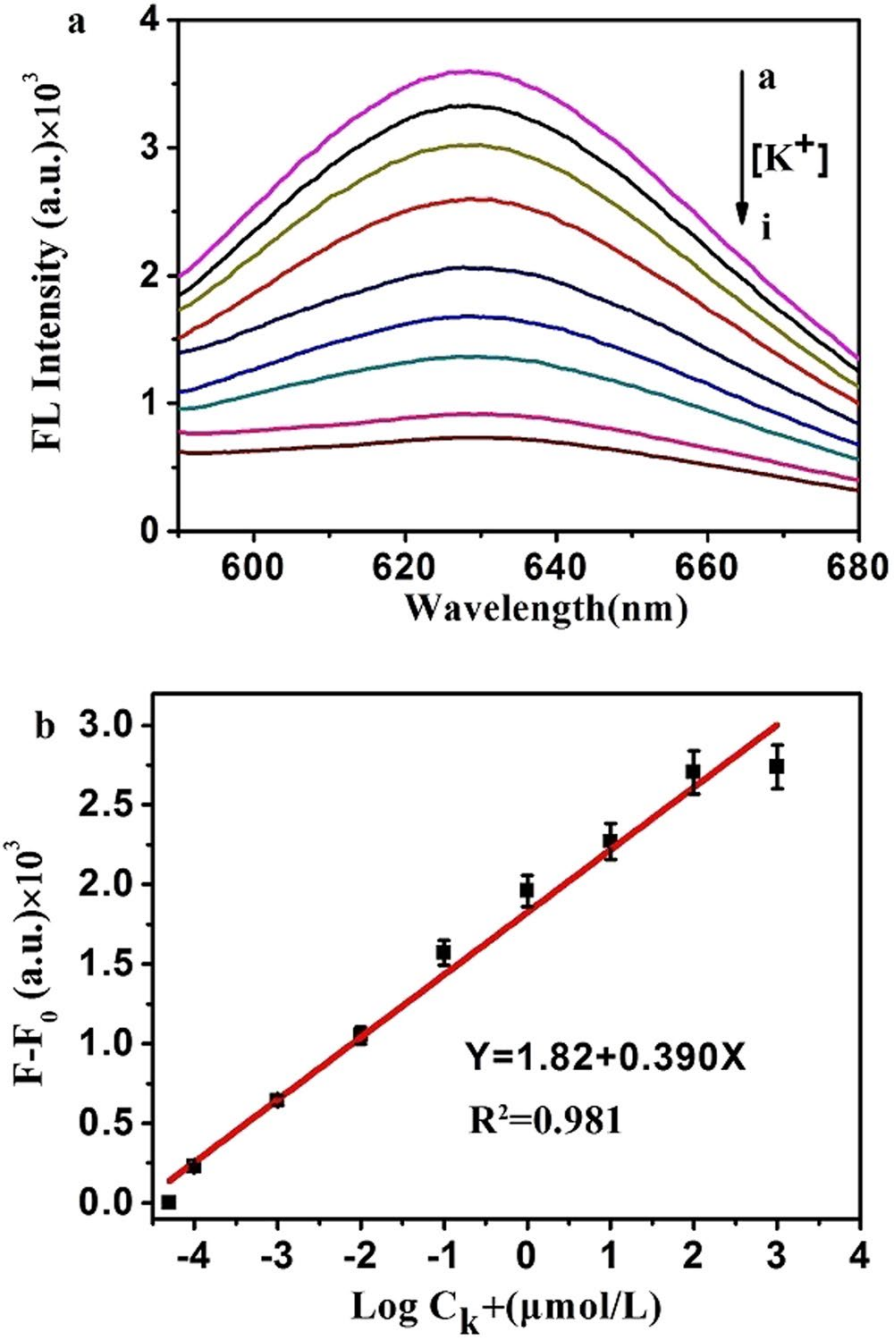
(**a**) Fluorescence spectra of DNA-AgNCs in the presence of different concentrations of K^+^ ions (from a to i), ranging from 0.1 nM to 1 mM. (**b**) Linear calibration curve between fluorescence intensity changes and the logarithm of K^+^ ions concentrations. F and F_0_ refer to the fluorescence intensities of the DNA-AgNCs system in the absence and presence of K^+^ ions, respectively.

Under the optimal conditions (pH 7.0 and 10 min of reaction time), the sensitivity and linearity of K^+^ ions detection using DNA-AgNCs fluorescence probes were carried out in different concentrations of K^+^ ions from 0.1 nM to 1 mM (Fig. 4a). It can be seen from Fig. 4b that the fluorescence intensity was decreased in the proportion with an increasing concentration of K^+^ ions. A linear calibration curve was attained between fluorescence intensity changes and the logarithm of K^+^ ions concentrations (R^2^ = 0.9810). The limit of detection for K^+^ ions was 0.1 nM, which was much lower than the concentration reported in the previous studies^31, 36^. Based on the DNA-AgNCs fluorescence sensing platform, the whole assay procedure can be completed in 10 min. All of these indicated that the proposed fluorescence method revealed sensitivities and rapidity to K^+^ ions assay in comparison with other detection systems.

### Selectivity of the fluorescence aptasensor

**Figure 5:**
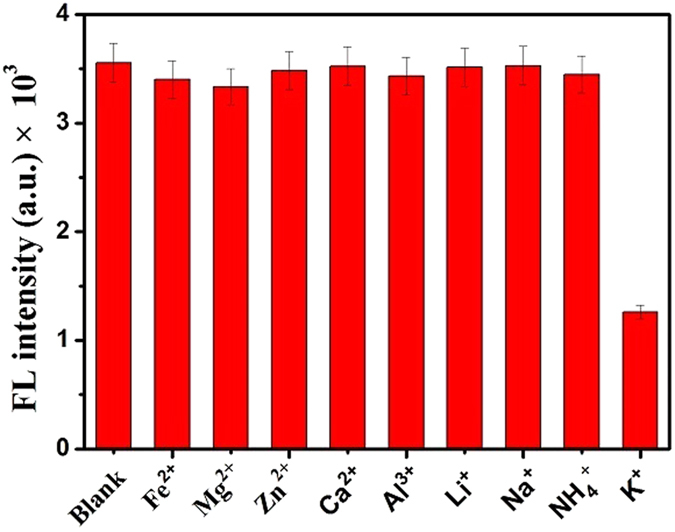
Response selectivity of fluorescence aptasensor toward K^+^ ions. The concentrations of Fe^2+^, Mg^2+^, Zn^2+^, Ca^2+^, Al^3+^, Li^+^, Na^+^, NH_4_^+^ and K^+^ ions were 10 μM.

The selectivity of this novel fluorescence aptasensor method was dependent on the excellent binding ability of aptamer toward the target K^+^. To explore the K^+^ sensing selectivity of this fluorescence method, eight kinds of common cation ions were measured in our detection system, including Fe^2+^, Mg^2+^, Zn^2+^, Ca^2+^, Al^3+^, Li^+^, Na^+^ and NH_4_^+^ (Fig. 5 **)**. The phosphate buffered solution without any other ions used as blank sample. The fluorescence values of DNA-AgNCs presented a slight decrease in the presence of these cations. As shown in Fig. 5, the results revealed that the fluorescence of DNA-AgNCs was strongly quenched and the fluorescence intensity decreased to 1258 a.u. after the addition of 10 μM K^+^ ions. However, the fluorescence intensity obtained upon addition of 10 μM Fe^2+^, Mg^2+^, Zn^2+^, Ca^2+^, Al^3+^, Li^+^, Na^+^ and NH_4_^+^ were about 3401 a.u., 3334 a.u. 3482 a.u., 3525 a.u., 3434 a.u., 3516 a.u., 3529 a.u., 3447 a.u., respectively. They are very close to the response value of blank sample (3556 a.u.), which indicated that these ions have a negligible quenching effect relative to K^+^ ions. Such a phenomenon meaningfully confirmed that the proposed method was highly selective for K^+^ ions and the fluorescence DNA-AgNCs probes revealed good biocompatibility between K^+^ ions and single-strand aptamers.

### Analysis of vitreous humour samples

**Figure 6:**
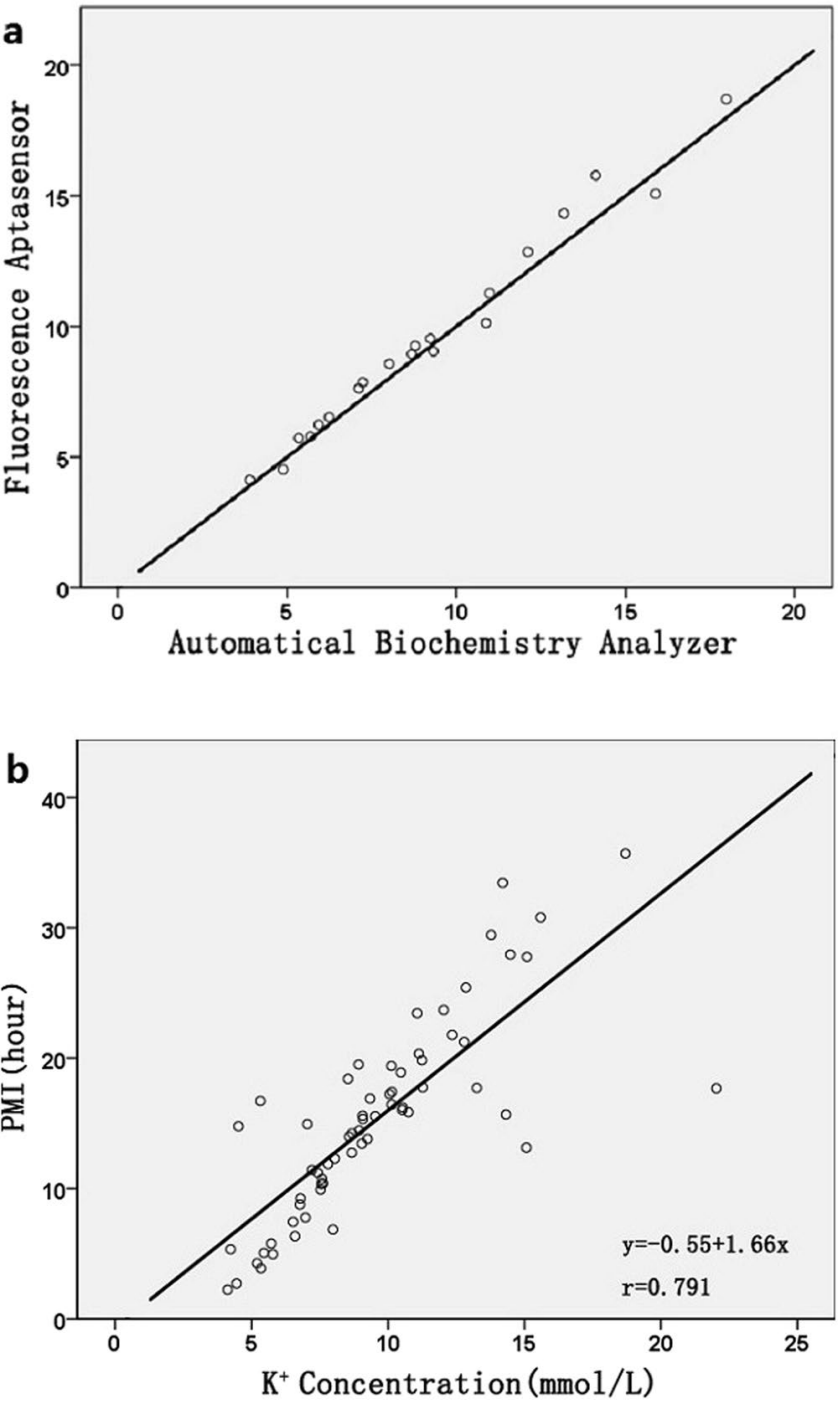
(**a**) Correlation between the fluorescence aptasensor and the automatic biochemistry analyzer. (**b**) Linear regression graph showing correlation between PMI and postmortem vitreous potassium.

After the fluorescence sensing method was developed, practical applications including determination of vitreous potassium and the PMIs estimation were studied. Considering that the fluorescence DNA-AgNCs sensing method was the first time to predict PMIs in medical-legal casework, we made further efforts to explore the application feasibility and accuracy of this proposed method. Twenty vitreous samples randomly were examined using both the fluorescence aptasensor and the automatic biochemistry analyzer (Fig. 6a). Obviously, as shown in the Fig. 6a, there was no significant difference between the fluorescence aptasensor method and automatic biochemistry analyzer towards the equation: y = x, which indicated that the proposed strategy held great promise in practical application.

In order to verify the vitreous potassium concentration that obtained by the proposed fluorescence sensing method can be used to estimate PMI, 63 VH samples from cases with known PMIs were investigated. VH samples were diluted to 1000 folds using PB buffer solution and the final vitreous potassium concentrations with range from 0 to 25 mM were calculated after fluorescence aptasensor analysis. Furthermore, a linear regression could be performed on the figures (Fig. 6b), and the equation for estimation of PMI was constructed: PMI(h) = −0.55 + 1.66 × C_K_^+^(coefficient of regression = 0.791). The results confirmed the strong relationship between vitreous potassium levels and PMIs, which has a great agreement with earlier studies^37, 38^. It was worth mentioning that there was a linear rise in vitreous potassium concentration with advancing PMI, which was first described by Sturner in 1963^2^. After Sturner’s original report, subsequent authors found a variety of linear or piecewise-linear relationship between K^+^ ions concentration and PMI^39–41^. The estimation of the confidence interval was based on the formulation of this corresponding study. Hence, the new formula in our study was necessary to attain trustworthy statistics for comparison between the known and predicted PMIs. In order to demonstrate the real significance of our research, 6 new cases with known PMIs were subsequently to evaluate the precision of the obtained formula, with the results shown in Table 1. According to Table 1, a good agreement between the values of PMI by our proposed method and known PMIs could be obtained (Absolute error ± 1.6 h), which indicated the reliability of our fluorescence sensing for detecting PMIs in real cases. Therefore, the developed fluorescent DNA-AgNCs probes can serve as an excellent sensing agent to quantitatively detect K^+^ ions in the real vitreous samples, and then K^+^ ions levels also could be successfully applied to evaluate the PMIs.

**Table 1:** Comparison of the estimated PMIs with the true PMIs.

Samples	Age	Cause of death	Known PMIs	Estimated [K^+^]	Predicated PMI	Absolute error
			T (h)		X (h)	E = X − T (h)
1	43	brain injury	24.0 h	15.69 mmol/L	25.5 h	+1.5 h
2	21	brain injury	12.0 h	6.72 mmol/L	10.6 h	−1.4 h
3	31	brain injury	19.0 h	10.99 mmol/L	17.7 h	−1.3 h
4	29	brain injury	23.8 h	13.77 mmol/L	22.3 h	−1.5 h
5	35	brain injury	25.0 h	15.75 mmol/L	25.6 h	+0.6 h
6	39	brain injury	24.7 h	14.25 mmol/L	23.1 h	−1.6 h

## Conclusions

In this work, a new fluorescence aptasensor strategy based on DNA-AgNCs probes has been developed for selective and sensitive detection of vitreous potassium. A novel DNA-AgNCs fluorescence probe was successfully synthesized using DNA template, where it has an aptamer for K^+^ ions. Under the optimal conditions, a linear range of 0.1 nM to 1 mM for K^+^ ions was found, with limit of detection of 0.06 nM. Moreover, the whole reaction for vitreous potassium sample does not need complicated pretreatment, only a centrifugation and a dilution are required. Relative to the existing methodologies, the fluorescence aptasensor method presented in the study has higher sensitivity and simpler operation in actual application. Furthermore, vitreous potassium concentration detected by the proposed fluorescence aptasensor method can be successfully used to estimate PMI. An equation between vitreous potassium and PMIs was obtained: PMI(h) = −0.55 + 1.66 × C_K_^+^(r = 0.791). Utilizing the vitreous potassium levels from 6 new vitreous samples, the PMIs can be estimated rapidly and accurately in terms of the linear equation, which may provide supporting evidence for forensic case investigations. Above all, the findings presented in this work demonstrated the possibility of the developed fluorescence aptasensor strategy to determinate vitreous potassium and following evaluate the PMIs, which offered a promising new detection approach in the forensic and clinical fields.
